# Understanding the Progression of Bone Metastases to Identify Novel Therapeutic Targets

**DOI:** 10.3390/ijms19010148

**Published:** 2018-01-04

**Authors:** Annie Schmid-Alliana, Heidy Schmid-Antomarchi, Rasha Al-Sahlanee, Patricia Lagadec, Jean-Claude Scimeca, Elise Verron

**Affiliations:** 1 Centre National de la Recherche Scientifique (CNRS), Université Côte d’Azur, Inserm, iBV, 06108 Nice, France; Annie.SCHMID-ALLIANA@unice.fr (A.S.-A.); Heidy.SCHMID-ANTOMARCHI@unice.fr (H.S.-A.); rasha.al-sahlanee@unice.fr (R.A.-S.); Patricia.Lagadec@unice.fr (P.L.); jean-claude.scimeca@unice.fr (J.-C.S.); 2 College of Sciences, Biotechnology Department, University of Bagdad, Bagdad, Iraq; 3 Regenerative Medicine and Skeleton. RMeS-Lab, INSERM UMR 1229, University of Nantes, 44000 Nantes, France; 4 Chimie et Interdisciplinarité, Synthèse, Analyse, Modélisation (CEISAM), UMR CNRS 6230, University of Nantes, 44300 Nantes, France; 5 Faculty of Pharmaceutical Sciences, University of Nantes, 44000 Nantes, France

**Keywords:** bone metastases, bone tropism, bone microenvironment, molecular mechanisms, therapeutic strategies

## Abstract

Bone is one of the most preferential target site for cancer metastases, particularly for prostate, breast, kidney, lung and thyroid primary tumours. Indeed, numerous chemical signals and growth factors produced by the bone microenvironment constitute factors promoting cancer cell invasion and aggression. After reviewing the different theories proposed to provide mechanism for metastatic progression, we report on the gene expression profile of bone-seeking cancer cells. We also discuss the cross-talk between the bone microenvironment and invading cells, which impacts on the tumour actions on surrounding bone tissue. Lastly, we detail therapies for bone metastases. Due to poor prognosis for patients, the strategies mainly aim at reducing the impact of skeletal-related events on patients’ quality of life. However, recent advances have led to a better understanding of molecular mechanisms underlying bone metastases progression, and therefore of novel therapeutic targets.

## 1. Introduction

Metastasis is the process through which cancer cells leave the initial tumour and travel throughout the body to establish a secondary tumour in other organs and anatomical sites.

Bone, is one of the most preferential metastatic target sites for cancers. Prostate, lung, breast, kidney, and thyroid primary cancers account for 80% of skeletal metastases [1]. Spine, pelvis, proximal femur, and skull are the most common sites of bone metastases.

Bone metastases can be divided into two broad categories; (i) osteolytic metastases that are associated with bone destruction and (ii) osteosclerotic metastases that are characterized by new bone formation. Bone metastases can be predominantly osteolytic or osteosclerotic, or mixed with features of both types [2,3].

Although precise molecular mechanisms underlying preferential cancer metastasis to bone need to be elucidated, it seems likely that bone provides an attractive environment that allows circulating cancer cells to home, survive and proliferate. This notion is consistent with the “seed and soil” theory proposed by Paget [4] in which he postulated that cancer cells, somehow liberated from the primary tumour, would seek nurturing conditions that resemble their original environment to grow and create a new lesion.

## 2. Seed and Soil Interactions

Bone metastasis occurs either via a direct invasion of bone tissue or secondarily to bone marrow, the latter being the most common. Bone marrow is colonized by metastatic cancer cells which in turn spread to the firm bone matrix [5]. In light of this, interactions of metastatic cancer cells (seed) with bone/bone marrow cells (soil) are critical for establishment and development of bone metastases [6]. This need for reciprocal interactions between cancer cells and the microenvironment make difficult to decipher mechanisms governing metastases formation, in particular the site specificity (i.e., preferentially in bones, lungs or liver for example) [6,7].

The binding of cancer cells at the metastatic site involves cell-to-cell interactions, or interactions between cells and the extracellular matrix, and these processes involves cell adhesion molecules like integrins [8]. It has been found that the endothelium represents the first point of interaction between metastatic cancer cells released in the blood stream and a specific tissue [9]. The success of this interaction is dependent on chemo-attraction and adhesion events according to different cell-surface markers, to morphological phenotypes and to specialized functions of the endothelium. For example, the presence of a stromal-derived growth factor (SDF-1—CXCL12) and its receptor CXCR4 on bone marrow endothelial cells has been shown to be favourable to cancer cell extravasation from the circulation and their retention in bone marrow [10]. This is particularly true for breast and prostate cancers, and a similar “seed and soil” interaction mechanism operating in the lung and liver may account for preferential formation of metastases within these environments [6].

The role played by this “seed and soil” interaction mechanism is clearly demonstrated by the inconsistency of metastasis formation throughout the skeleton. Indeed, the axial skeleton represents a preferential target, in accordance with the haematopoietic bone marrow content, while almost no metastases develop within hands and feet where no haematopoietic bone marrow is present [4,11].

## 3. Theories of Metastatic Progression

Two main models of metastatic progression are currently accepted. The classical model is a linear progression model, and the more recent one is the parallel progression model that represents somewhat of a paradigm shift [12].

In the linear progression/late dissemination model, a normal epithelial cell acquires mutations that inactivate tumour suppressor genes and/or activate oncogenes, which promotes survival and uncontrolled proliferation of cancer cells. An increased proliferation rate may also lead to mutations in progeny cells, which result in a variety of subsequent mutations heterogeneously distributed throughout discrete tumour sub-populations [13,14,15]. Some of these mutations are associated with gene alterations that facilitate dissemination and invasion, and these mutated cells are capable of generating distal metastases. In this model, acquisition of malignant mutations is linked to tumours of large size and occurs at later stages of tumour growth [16,17,18].

The second model, is known as the parallel progression/early dissemination model [19] and has gained favour recently, although it dates from the 1950’s [20]. This model rejects the notion that tumours must reach a defined size before acquiring the ability to disseminate, and it posits that dissemination occurs long before detection of the primary tumour [21,22,23]. Nevertheless, both models agree that cancer progression depends on clonal expansion of “fittest” cells. The idea that tumour cells disseminate early is supported by the notion that in rare cases, patients with very small early-stage primary tumours (T1) present metastases at the time of diagnoses. This concept is further sustained by the fact that metastatic cancers of unknown primary origin represents 5–10% of all cancer diagnoses in the United States and Europe [24].

There is a third recent theory, known as the “pre-metastatic niche”, which is based on observations made during the last decade [25]. This theory has radically changed our view of metastatic colonization. It holds that secreted factors (S100A8, S100A9, osteopontin OPN) are produced either by the primary tumour or by stromal cells in the metastatic site, which serve to “prime” tissues for later metastatic tumour engraftment [17,26,27]. These factors facilitate mobilization and recruitment of bone marrow-derived cells that can liberate pro-inflammatory cytokines such as TNF-α. These inflammatory cytokines “prime the soil” by activating endothelial cells and inducing matrix metallo proteins (MMPs) expression to promote subsequent cancer cell colonization and outgrowth [2].

A key observation in the field of cancer metastasis is that specific cancers tend to colonize particular sites. For example, breast cancer most commonly metastasizes to bone, followed by lung, brain, and liver [28], whereas colorectal cancers most frequently metastasize to liver, then lung, and rarely to bone and brain [29,30]. Stephen Paget first offered in 1889 an explanation for this observation. His seminal “seed and soil” hypothesis suggested that to support the growth of metastases, the “soil” (i.e., metastatic environment) must be capable of nourishing the “seed” (i.e., a disseminated cancer cell) [4,30,31]. For instance, the bone microenvironment may provide sufficient growth signals to facilitate breast cancer outgrowth while these same signals are less effective at promoting colon cancer outgrowth. This hypothesis was challenged nearly 40 years later when James Ewing claimed that differences in metastatic colonization by certain cancers could be explained by anatomy of the local circulatory system [32]. Thus, cancers will rather metastasize to secondary sites presenting the highest degree of accessibility via the vasculature and blood flow patterns [31,33]. This is best exemplified by the high propensity of colon cancers to metastasize to liver [34], which is the first organ they encounter following intravasation into the portal circulation. It was later shown that while local recurrences correlated with increased perfusion rates, the formation of distant metastases could not be completely explained by anatomical vascular access patterns, and Paget’s “seed and soil” hypothesis prevailed and remained a guiding principle in the study of organ-specific metastatic process. 

## 4. Gene Expression Profiling of Bone Metastases

In recent years, gene expression profiling has become a standard technique used to identify genes that are deregulated in cancer. A better understanding of the various stages of metastatic progression has been gained by coupling gene-expression profiling with pre-clinical mouse models of breast cancer metastasis to bone. Some of these approaches, starting from heterogeneous cultures of breast cancer cells, involve derivation of sub-populations that preferentially spread to bones. These cell populations are isolated directly from established bone metastases that have been obtained following injection of the parental population into the left cardiac ventricle or the mammary fat pads of mice. Successive rounds of in vivo selection result in breast cancer cells that aggressively metastasize to bone tissue when compared to the original cell population. These approaches have facilitated the identification of individual molecular mediators of metastatic process such as IL-8 [35], as well as sets of genes that work cooperatively including *CXCR4*, *MMP-1* and the TGFβ-regulated genes *CTGF* and *IL-11* [36,37,38,39]. On the other hand, the in vitro isolation of single cell progeny (SCP) yielded breast cancer cell-derived populations with vastly variable in vivo bone metastatic phenotypes [40]. Importantly, compared with aggressive bone metastatic cell populations that were derived by in vivo selection, aggressive bone metastatic SCPs present gene-expression profiles that are largely overlapping [16]. These studies, in addition to identifying novel mediators of metastasis, provide insights into the nature of the metastatic process. 

## 5. Bone Metastasis Microenvironment

Bone is a unique microenvironment made of proteins and calcified hydroxyapatite crystals forming a dense matrix that is tightly interconnected to bone marrow, which contains osteoblast and osteoclast progenitors, as well as hematopoietic stem cells (HSCs). Bone marrow is a major site of metastatic diseases for breast and prostate carcinomas, and for multiple myeloma [25,41]. Although metastasis formation in lungs, liver and bones displays some similarities, the bone microenvironment undergoes constant remodelling events that deeply impacts metastasis onset and development [42]. This remodelling process results from an equilibrium between osteoblasts and osteoclasts activities, which are regulated by mechanical stress, cytokines and hormones. Should this balance be disturbed, osteolytic or osteosclerotic metastases appear, depending on the predominant activity occurring within lesions [5,43,44].

### 5.1. Osteolytic Bone Metastases

Osteolytic lesions arise when osteoclast-mediated bone resorption overcomes bone formation by osteoblasts, resulting in weakened structures that compromise bone integrity [5,6]. Various factors including cytokines and hormones control the bone remodelling equilibrium [5], and a vicious cycle involving osteoclasts and tumour cells progressively leads to the development of osteolytic lesions. Briefly, the production of parathyroid hormone-related peptide (PTHrP) and the expression of receptor activator of nuclear factor—κB receptor ligand (RANKL) play important roles in osteolytic metastases, and in bone metastases arising from primary breast cancer. It has been observed that PTHrP expression was significantly higher compared to metastases from other primary cancers [11]. PTHrP increases RANKL expression, which acts on osteoclasts to increase osteoclast maturation and resorptive activity. Subsequently, the resorptive activity releases TGFβ and other growth factors like EGFs or IGFs from the bone matrix [5,45,46], generating a feed-back loop that stimulates metastatic proliferation [45]. Once released, TGFβ in turn stimulates PTHrP production, which is further increased by a high local calcium concentration resulting from bone resorption [47,48]. Moreover, TGFβ regulates osteolytic and pro-metastatic agents, as well as other microenvironment factors like hypoxia [49], which promotes the growth of tumour cells. Indeed, TGFβ activates the epithelial to mesenchymal transition (EMT) [50], increases tumour cells invasiveness and angiogenesis, and displays also immunomodulation properties [51].

In addition, stromal cells present within the bone marrow microenvironment are involved in the establishment and the progression of bone metastases. These includes neurons, blood platelets and endothelial cells. Thus, sympathetic neurons activation by bone metastasis is responsible for severe pains [52], but also for increasing tumour proliferation and invasiveness [53]. On the other hand, tumour cells bind preferentially to endothelial cells and activate platelets aggregation, which induce angiogenesis and increases tumour survival and proliferation [54]. Indeed, platelet aggregation initiates a massive production of lysophosphatidic acid (LPA), which acts as a pro-metastatic lipid mediator that promotes survival, proliferation, motility and invasiveness of breast cancer cells for example [55]. Likewise, via the activation of G-protein-coupled receptors, LPA acts directly on tumour cells by stimulating the secretion of pro-osteoclastic interleukins such as interleukins (IL) IL-6 and IL-8 among others [56,57].

Concerning IL-6, it increases bone degradation via: (i) the production of RANKL, and the negative regulation of osteoprotegerin (OPG); (ii) the induction of proteins involved in bone resorption such as PTHrP, interleukin (IL) IL-8, IL-11 and Cox-2; (iii) an increase of oestradiol 17β-hydroxysteroid dehydrogenase activity (an inhibitor of the anti-osteoclast activity of oestrogens); (iv) the stimulation of DKK-1 expression by tumour cells and (v) the downregulation of collagen II and aggrecans by the osteoblasts [31]. Interestingly, these last two functions of IL-6 illustrate that the bone loss observed in osteolytic metastases is also partially due to the failure of osteoblasts to produce new osteoid and repair the bone matrix. In addition, other factors that play a role in osteolytic bone metastases are secreted or induced by metastatic tumour cells (IL-1, prostaglandin E2, granulocyte macrophage colony stimulating factor GM-CSF, tumour necrosis factor-alpha TNFα, matrix metallo protein MMPs, cathepsin K CTSK and osteopontin OPN) [54,58,59,60,61,62,63].

Finally, as already mentioned above, osteolytic lesions are not only the result of bone resorption mediated by osteoclasts. Indeed, they are also due to a decreased bone formation mediated by osteoblasts, and the antagonistic expression of Wnt and bone morphogenetic proteins (BMP) could play an important role [64,65]. Consequently, the bone metastatic niche favours a complex vicious cycle comprising interconnected processes, which feed on each other, that is bone metabolism regulations and tumour growth.

A good understanding of these mechanisms and pathways is crucial to develop therapeutic strategies, and several pharmacological inhibitors are already being tested in clinical trials [11,46,66,67].

### 5.2. Osteosclerotic Bone Metastases

Osteosclerotic bone metastases result from excessive bone formation, which produces often a disorganized and weak bone tissue. This imbalance is mostly encountered with metastases originating from prostate and breast primary tumours [5,6,29,66]. However, in contrast to osteolytic bone metastases, the mechanisms involved are less understood. The most influential factors so far identified are BMP and endothelin-1 (ET-1) [5,6,29]. The up-regulation of BMP expression by osteoblasts proportionally affects bone formation, and it is also involved in ectopic bone formation [6]. Compared to other primary cancers, BMP expression is high in cancerous prostate cells, correlating with the high occurrence of osteosclerotic metastases observed for primary prostate cancers [2]. Concerning ET-1, it is a potent vasoconstrictor, a direct stimulant of osteoblast progenitors mitogenesis, and it has been linked to the substantial pain resulting from osteosclerotic lesions [5,6,29].

**Figure 1:**
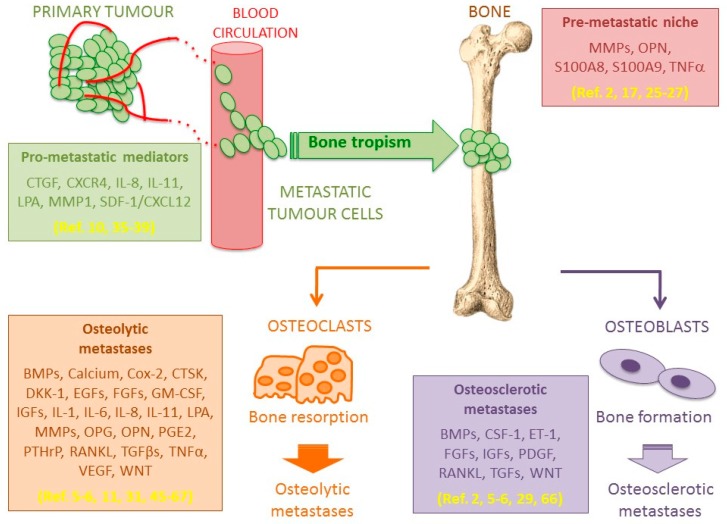
Factors involved in hematogen dissemination and tissue invasion by cancer cells with a bone tropism. Factors involved in bone metastatic progression are mainly related to (i) migration of metastatic tumour cells within blood circulation; (ii) invasion of bone marrow niche; and (iii) development within bone tissue through two mechanisms leading to osteolytic or osteosclerotic lesions, both leading to severe skeletal-related events.

Figure 1 summarizes the main steps of bone metastasis progression from the primary tumour to the development of osteolytic or osteosclerotic lesions.

## 6. Bone Metastasis Treatments

Patients bearing bone metastases often suffer from many complications including pain, decreased mobility, neurologic compromise and pathologic fractures [68]. These complications, generically called skeletal-related events (SREs), impair the patient’s prognosis and increase the cost of treatments.

Despite numerous studies attempting to better understand bone metastasis pathophysiology, there are as yet no established and efficient clinical methods for their cure and/or their prevention. Currently, medical management of bone metastases is based on local approaches (i.e., surgery, radiation therapy) or systemic strategies such as molecular targeted therapies [66,69].

Depending on the case, local treatment of bone metastases can be done either by a consolidation procedure and/or tumour destruction by using surgery, irradiation (stereotactic) or interventional radiology techniques (i.e., vertebroplasty, radiofrequency, cryoablation).

### 6.1. Ablative Procedures

Local treatments of bone metastases have a dual role: (i) mechanical stabilization to prevent pathological fractures; (ii) tumour destruction to block or slow tumour and, for example, to fight against neurological complications in case of spinal metastases. It is noteworthy that in almost 90% of cases, ablative treatments lead to a relief from pain within 24 h [70].

#### 6.1.1. Surgical Techniques

Surgical approaches are generally used for patients with impending or complete pathological fracture, or for patients exhibiting spinal cord instability or compression. Indeed, surgery may represent an emergency solution in the case of compression, a situation which requires the restoration of skeletal integrity to limit neurological impact. Concerning extra spinal bone metastases, about 92% of surgical procedures result from complete or imminent fractures [71]. 

#### 6.1.2. Irradiation Techniques

Ablative irradiation with tumouricidal doses are used for bone metastases limited in size and number (1 to 3 metastatic sites), and/or with a vertebral localisation close to the spinal cord. In 50 to 80% of cases, radiotherapy is used as an analgesic treatment, with the aim of getting an immediate relief, preventing complications, and improving the quality of life of patients. Most of the studies reported in the literature are related to the spine, while information concerning other metastatic sites (limbs, ribs) is limited [72,73].

#### 6.1.3. Thermal and Chemical Ablation

Interventional radiology uses several techniques (cementoplasty, radiofrequency ablation, cryotherapy, chemoembolization) to achieve bone consolidation with varying degrees of tumour cells destruction [74,75].

- Radiofrequency ablation - Radiofrequency ablation of bone metastases is a recent radiological percutaneous technique, using alternative current (450–600 kHz) applied through a needle entered in the metastasis under radioscopic control. Radiofrequency energy generates an overheating (60–70 °C), and cancer cells necrosis is obtained by heat destruction within a volume of 3 cm in diameter. This technique cannot be used in the close vicinity of nerve tissues, since they do not support a temperature above 45 °C. It is also difficult to implement this technique for metastases affecting flat bones (i.e., sternum, iliac wing). Radiofrequency can also be used in combination with cementoplasty, although this combination has not yet shown significant improvement compared to cementoplasty alone [72,74].
- Cryotherapy—Cryotherapy is the more recent technique that enables tumoral destruction, over areas up to 5 cm, by low temperature using a needle-like applicator and liquid argon. This technique involves generally little pain and it can be realized under simple sedation, contrary to radiotherapy that usually requires general anaesthesia. On the other hand, cryotherapy cannot be combined with cementoplasty since tissue cooling exerts an inhibitory effect on cement polymerization [72,74].
- Chemoembolization—Chemoembolization consists of delivering directly to tumours an antimitotic infusion or antimitotic-loaded microparticles (carboplatin and adriamycin). This technique is used to treat bone lesions that have been previously irradiated, or which are unresectable and resistant to other treatments. In 50% of cases, a partial or complete response is observed, and this method is often very effective in treating initial metastases (especially from breast cancer) [72,74].

**Figure 2:**
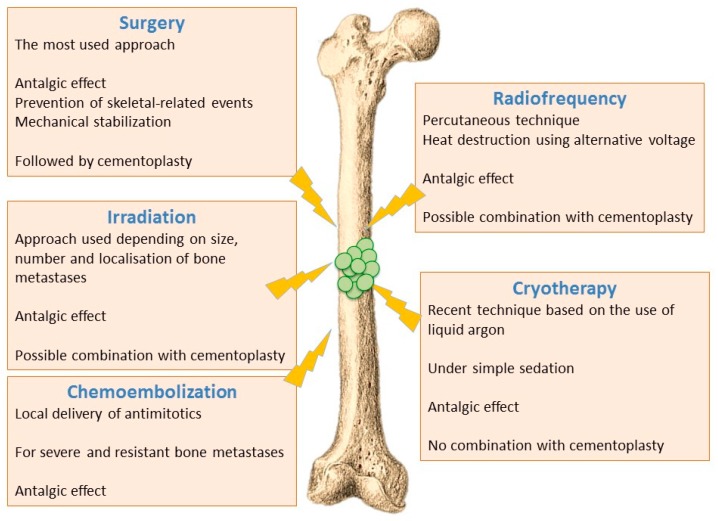
Tumours ablative therapies. They are selected according to the clinical status of patients, the tumour resistance, characteristics of metastases in terms of size/number/localization, and therapeutic objectives such as preventing development of skeletal-related events, achieving analgesic effect and getting a mechanical stabilization (see references [70,71,72,73,74,75]).

Figure 2 recapitulates the main ablative procedures proposed to patients.

### 6.2. Reconstructive Procedures

Cementoplasty, which is the simplest and most suitable technique to obtain vertebral body stabilization, represents the most common interventional radiology technique. It consists of injecting percutaneously, under radiologic control, a surgical cement in bone lesions (mostly PMMA, poly (methyl methacrylate)). In almost 90% of cases, mechanical stabilization and analgesic effects are obtained. In addition to these effects, the in situ recurrence rate is low (around 14%). This phenomenon can be explained by heat generation induced by the cement polymerization (60–70 °C), and/or by a direct toxic action of cement monomers on tumour cells [76]. In addition to osteolytic metastasis treatment, cementoplasty can also be efficient in the context of osteosclerotic or mixed metastases. However, in these circumstances, the technical implementation is more complex and therefore, a high rate of postoperative complications is observed. Cementoplasty can be applied in numerous sites including spine, humeral head or intertrochanteric region. However, some locations remain out of the scope of this technique (femoral neck, parts of the hip bone), especially if there is an associated fracture that requires a surgery procedure [72,75].

These procedures may also involve an endoprosthetic reconstruction or consolidation techniques using intramedullary nails or metallic equipments such as plates and screws [77].

### 6.3. Pharmacological Approaches

One has to keep in mind that systemic administration of chemotherapeutic agents may alter the normal skeletal development and bone remodelling, leading to increased risks of fractures [78]. Considering the complex set of interactions between metastatic cells, the microenvironment, and bone-resident cells, therapies cannot be based on the genetic profile of primary tumours. In this context, the concept of vicious cycle has changed the therapeutic approach, and bone resorption-blocking strategies have emerged as new potential treatments [79], together with several other pharmacological approaches including therapies targeting cancer cells and tumour vascularization [80].

#### 6.3.1. Bisphosphonates

Due to their chemical and pharmacological properties, bisphosphonates (BPs), which have been initially developed for the treatment of osteoporosis, are also indicated for treatment of bone metastases [81].

Due to their chemical structure, BPs display a strong affinity to hydroxyapatite crystals forming the mineral phase of the bone matrix. They are synthetic products derived from inorganic pyrophosphates (PPi), in which the P-O-P bond is replaced by a P-C-P bond to increase the chemical resistance to enzymatic cleavage. The phosphonates groups can chelate calcium ions present within biological hydroxyapatites, and this affinity increases when R1 is a hydroxyl group [81]. Accordingly, two models of bonds have been presented: (i) a weak bond, corresponding to one single phosphonate group (etidronate); (ii) a stronger bond carrying two phosphonates and hydroxyl groups, (alendronate, zoledronate). Recently, it has been suggested that the R2 group could also impact BPs’ affinity for hydroxyapatite crystals. Finally, it has been shown that, compared to compounds with a higher affinity, low-affinity molecules can penetrate more deeply within the mineral matrix, and so doing could reach the canalicular network [81].

Concerning the mechanisms of action, BPs’ affinity decreases in acidic conditions (resorption lacuna) because of phosphonate-groups protonation. Once they are released from the bone matrix, BPs can be internalized into osteoclasts through endocytosis. Afterward, via the inhibition of the farnesyl diphosphate synthase, BP prevent the prenylation of several molecules (protein G trimeric subunits, phosphodiesterase subunits) involved in many cellular processes crucial for osteoclastic functions (cytoskeleton architecture, actin rings formation, wrinkling of the border, intracellular vesicles movements). The fusion of osteoclastic precursors is still possible and leads to multinucleated giant cells, which are inactive. At high concentration, BP can induce osteoclasts apoptosis. In all cases, by inhibiting bone resorption, they deprive tumour cells of growth factors released from the bone matrix upon osteoclastic action (TGFβ, IGFs) [81].

Much preclinical evidence suggests that BPs possess direct antitumor properties [82,83,84]. After internalization, BPs significantly disrupt the function of small GTPases proteins by blocking their prenylation, which is essential for adhesion, migration, invasion and proliferation of tumour cells. Similarly, it has been suggested that BPs could inhibit αVβ3 integrin activation, which is involved in tumour cells adhesion on the bone surface [85]. Moreover, BPs could induce tumour cell apoptosis through cell structure alteration and DNA fragmentation [86].

BPs could have also an indirect antitumor activity by blocking angiogenesis. Actually, BPs regulate the proliferation, the migration and the adhesion of endothelial cells, and they inhibit the development of capillaries. Finally, BPs could have an indirect antitumor action via the modulation of the immune system through the activation of γδτ T lymphocytes [87]. This activation is caused by: (i) structural similarity between BPs and non-peptidic ligands that activates γδτ T cells; (ii) the recognition by the TCR (T cell receptor) of isopentyl pyrophosphate accumulated after the inhibition of farnesyl diphosphate synthase [81,82,88,89].

BPs (clodronate, pamidronate, ibandronate, zoledronate) are effective for the prevention and the retardation of SREs in patients with bone metastases from solid tumours or with multiple myeloma osteolytic lesions. Interestingly, several generation of compounds are available [90]. It has been shown that N-BPs of the second and the third generation (nitrogen-containing BP: zoledronate, palmidronate, alendronate, ibandronate) are more efficient in reducing SREs related to bone metastases coming from solid tumours other than breast and prostate cancers [91] or from castration-resistant prostate cancers [92].

BP administration is proposed for all patients with bone metastases from solid tumours or with bone lesions from multiple myeloma [93]. Similar to the strategy used for the patients with breast cancers, BP therapy should be initiated as soon as the discovery of X-ray evidence of bone destruction, or abnormal bone scan images combined with localized pain, and it should be continued throughout the disease [94]. In other words, BPs should be started at the first diagnosis of bone metastases [95].

Effectiveness of BP therapies requires a high level of patient compliance. However, this can be compromised by the extended duration of treatment, the use of another treatment, or private health insurance concerns in certain countries. Oral administration of BPs often triggers gastrointestinal side effects, and it requires strictly controlled administration conditions. In particular, patients must stay in a vertical position up to 2 h after drug intake, and they are advised to avoid food that could interact with BP metabolism. Concerning intravenous (iv) administration of BP, it is associated with development of symptoms similar to flu, principally after the first administration. In some cases, and following IV administration of high doses, severe side effects such as osteonecrosis of the jaw have been reported [81,96,97].

#### 6.3.2. Denosumab

Denosumab is a human monoclonal antibody developed for the treatment of osteoporosis and bone metastases [78,98,99]. Denosumab therapy reduces and delays the risk of SREs in patients with bone metastases from breast cancer, prostate cancer, non-small cell lung cancer and other types of solid tumours. Indeed, it binds and neutralizes RANKL, similarly to the decoy receptor OPG that blocks RANKL receptor activation on the surface of osteoclasts and their precursors, preventing osteoclastogenesis, as well as osteoclasts activity and survival [95]. As a result, bone resorption is decreased and the tumour-induced bone destruction is inhibited.

Denosumab is administered by subcutaneous injections, which provides an alternative route of administration and eliminates the need for routine test of renal function prior to administration [100]. Hence, its use is widely recommended for patients with renal failure or receiving a nephrotoxic chemotherapy like cisplatin [101]. Regarding long-term impact, a study in postmenopausal women with low bone mass and treated with denosumab reported that stopping the treatment resulted in decreased bone density at lumbar spine and hip sites within 12 months following the final dose [102]. Since denosumab use is recent, no data are available about patients’ compliance. However, observance should be better compared to treatments with BP. Indeed, good tolerance is reported, and the administration mode is more adaptable and less restrictive [81,99].

#### 6.3.3. Other Agents Targeting Bone Tissue

Therapeutic strategies targeting osteoclastic bone resorption represents an area of intense clinical research [81]. This includes inhibitors of RANK/RANKL interaction (osteoclasts formation), as well as Ctsk and αVβ3 integrin inhibitors [89,103]. Interestingly, these therapies also impact on tumour cells and on stromal components involved in bone metastatic disease [57].

The RANK/RANKL/OPG triad appears as a key player in tumour expansion within bone tissue. Indeed, RANKL level is increased in osteolytic lesions related to malignant tumours, while OPG level is increased in osteosclerotic lesions [104]. In animal models of bone metastases from breast cancer, OPG inhibits bone destruction and reduces bone tumour mass. This reduction of tumour development is probably related to the inhibition of osteoclastic resorption, since OPG does not affect tumour growth in soft tissue. In addition, RANK-related RANKL inhibitors (Fc-OPG) [105], neutralizing antibodies directed against RANKL, and soluble antagonists of RANK (Fc-RANK) are inhibitors of bone metastases, as observed in preclinical and clinical models [80,106].

The use of the semi-metallic element gallium (Ga) offers promising prospects. Indeed, through its action on osteoclastogenesis, Ga is an inhibitor of bone resorption [107]. Moreover, previous experiments have established that Ga would represent an attractive additive to calcium phosphate-based biomaterials for bone reconstructive surgery [108]. Ga’s direct effects on tumour cells have been recently studied in the context of bone metastases. The in vitro data obtained suggest that, through its action against the vicious cycle involving bone cells and tumour cells, Ga represents a relevant and promising candidate for a local delivery upon the resection of bone metastases from breast cancer [109].

As new potential diagnosis and therapeutic markers for bone metastases, microRNAs (miRNAs) are also currently investigated. During the last years, the list of miRNAs involved in the progression of breast and prostate cancers has grown, including those directly involved in cancer cells metastasis to the bone [110]. Analysis of osteoclasts-derived miRNAs that are induced in the presence of tumour cells also led to the discovery of specific miRNAs (miR-16, miR-378, miR-141) that might have an influence on osteolytic bone metastases [111,112,113]. As another example, MiR-34a is considered as an anti-osteoclastogenic factor negatively regulated by transforming growth factor-β-induced factor 2 (Tgif2) [114]. By consequence, Tgif2 deletion may reduce bone resorption by abolishing miR-34a regulation.

Several strategies targeting TGFβ signalling pathways are under study in the context of cancer therapies. The different classes of TGFβ inhibitors used in preclinical models and clinical trials include: (i) monoclonal antibodies targeting TGFβ and preventing TGFβ interactions with its receptor; (ii) small molecules that inhibit TGFβRI kinase activity and TGFβRII-mediated activation of TGFβR1-SMAD proteins; (iii) a naturally derived product, halofuginone (HFG), which inhibits TGFβ [115]. Because TGFβ signalling pathways are activated in numerous cell types, the blockade of the TGFβ axis could have adverse effects on wound healing and on the immune system. Nevertheless, clinical studies (patients with advanced metastatic melanoma, colon cancer, prostate and breast cancers) have shown that a small inhibitory molecule (LY2157299) was well-tolerated and displayed minimal toxicity [116].

Considering the variety of factors released from the bone matrix upon osteoclastic resorption (IGF1, PDGFs, BMP, calcium etc.), and which stimulate the growth of certain tumours, this field of research represents also a potential source of pharmacological targets capable of interfering with metastatic processes [57].

Endothelin 1 (ET-1) is one of the promising osteoblastic targets studied in preclinical and clinical trials. This vasoconstriction agent stimulates osteoblastic proliferation and differentiation, and it is involved in the development of skeletal metastases of prostate cancer [117]. Therefore, atrasentan was the first receptor antagonist to ET-1 sub-type A studied in patients with prostate cancer, while zibotentan, a more selective inhibitor, is currently used in clinical trials [118].

Bone stroma and the osteoblastic niche can interact with cancer cells and thus promote a resistance to cytotoxic chemotherapy. Cancer cells are often resistant because they are maintained on the G0 phase of the cell cycle by contact with stromal cells present within bone marrow. A new therapeutic approach exploits the fact that tumour cells use CXCR4 and VLA4 proteins for bone marrow homing [119]. In addition, the use of mobilizing agents for MSCs (AMD3100, VLA4 target agents), which drive leukaemia and melanoma cells out of bone marrow, leads to a better response to chemotherapy in animal models. This approach is under investigation in clinical trials for the treatment of acute myeloid leukaemia and multiple myeloma [57].

#### 6.3.4. Agents Targeting Tumour Cells

As mentioned above, bone metastases result from selection and enrichment of pre-existing cellular subpopulations displaying gene expression signatures associated with metastatic bone development, (*CXCR4, MMP-1, FGF5, CTGF, IL-11, OPN*, follistatin, proteoglycan 1). Identification of these genes, and the corresponding signalling pathways, could provide potential therapeutic targets. Indeed, anti-CXCR4 antibodies reduce significantly the number of bone metastases in a model of prostate cancer, and the blockage of CXCR4 expression by siRNAs (small interfering RNAs) decreases in vitro the invasive character of breast cancer cells and inhibits the metastatic phenomenon in an animal model [120].

Growth factors and cytokines signalling pathways protect metastatic cells from anticancer drugs-induced apoptosis. Thus, targeting these survival factors in bone (TGFβ, IGF, IL-6, FGF, ET-1 and others) could increase cancer cells sensitivity to chemotherapeutic agents. 

Identification of metastases suppressor genes (MSGs:) also provides new insights into the formation of bone metastases. These genes block the metastatic capacity without impacting growth of the primary tumour. For example, the recovery of PTEN expression (Phosphatase and Tensin homolog) inhibits the development of bone metastases from prostate cancer, while it has no effect on lung metastases [121].

In parallel to MSGs, miRNAs have been identified as another class of molecules which regulates negatively the formation of bone metastases. This is the case of miR-335 and miR-126, which have been identified as metastasis suppressors in breast cancer [80,104].

#### 6.3.5. Agents Targeting Angiogenesis

Antiangiogenic treatments appear as attractive and promising anticancer therapies, but they have been less studied in the bone context. Indeed, to reduce the blood supply to a tumour is a challenge since bone marrow is a highly vascularized site, which can be strongly affected by antiangiogenic inhibitors [80]. In bone metastasis models, the antiangiogenic agent avastin inhibits tumour growth via indirect targeting to osteoclasts [122]. On the other hand, the exact target of antiangiogenic therapies is unknown, since actions of such agents on circulating endothelial progenitors have not been clearly established. However, the combination of an antiangiogenic agent with a cytotoxic chemotherapy has given promising results in an animal model of bone metastases from breast primary tumour [123].

Endothelial progenitor cells derived from bone marrow represent potential targets of antiangiogenic strategies. The difficulty here lies in the absence of consistent markers for endothelial progenitors that could be used to target them.

MTA1 (metastasis-associated protein 1) is a proangiogenic factor involved in prostate cancer progression [124]. Accordingly, silencing MTA1 effectively limits the expression of proangiogenic factors like vascular endothelial growth factor (VEGF) by prostate cancer cells, and represents thereby a new potential target for patients with prostate tumour [57].

#### 6.3.6. Agents Targeting Pain

Bone pain is very difficult to treat and tends to be resistant to opioids [125]. Indeed, bone tissue is densely innervated by primary afferent neurons and sympathetic neurons located in the intramedullary bone and the periosteum. Bone resorption inhibitors decrease substantially the pain caused by expanding bone metastases. Honore et al. demonstrated that osteoclastic resorption triggers recruitment of nervous fibbers related to pain and leads to bone pain-inducing modifications of spinal cord neurochemical properties. OPG, an antagonist of RANKL, prevents activation of nerve fibbers associated with pain, and reduces pain behaviours in mice with bone metastases [126]. In addition, tumour cells residing within bone secreting factors (NGF nerve growth factor, ET-1) activate neurons-associated pain perception. Therefore, bone resorption inhibitors, receptor A of ET-1 and NGF represent therapeutic targets for bone pain treatment. Another approach for patients with bone metastases is the use of radiopharmaceutical molecules (i.e., strontium, samarium and radium) that have a high avidity for calcified bone matrix. Finally, these agents have a slight antitumor effect via localized radiations, but they have substantial effects on bone pain and hence they are principally indicated because of their analgesic action [57,127].

## 7. Conclusions: Immunotherapy Perspectives

Identifying chemokines and their receptors involved in cancer biology is of growing interest due to their involvement in multiple steps of the metastatic process including proliferation, extravasation, survival of tumour cells and tumour angiogenesis [128,129,130,131,132]. Chemokines have been shown to control angiogenesis: (i) directly through interactions with their receptors expressed on endothelial cells, as described for CXCL5, CXCL8 and CXCL12; (ii) indirectly through the recruitment and the stimulation of leukocytes resulting in the production of angiogenic factors [133,134]. Lastly, tumour invasion is also controlled by chemokines and their receptors and among them, CXCR4 is the most frequently overexpressed receptor [135].

Interestingly, through their ability to recruit many leukocyte sub-populations, chemokines and their receptors regulate also the immune response toward the tumour [82,136,137,138,139]. Understanding these mechanisms provides numerous molecular targets for potential therapeutic applications. Several types of therapy can be explored including blocking interactions between chemokines and their receptors, interfering with receptor transduction pathways, or providing a local delivery of chemokines able to trigger an anti-cancer immune response [140,141,142]. In that respect, Bidwell et al. identified an innate immune pathway (Irf7 pathway) intrinsic to breast cancer cells involved in the development of metastasis. Based on this result, they suggested that the restoration of Irf7 may reduce bone metastases [143]. 

However, despite the development of these novel therapies, few studies address specifically the issue of bone metastases treatment, and this research area will certainly continue to expand. Indeed, unlike ‘’passive’’ strategies involving the use of antibodies, such innovative and ambitious therapies can be considered as ‘’active’’, since they trigger the immune system response against cancer cells, including an immune memory resulting in long-term benefits.

## Abbreviations

BMP	bone morphogenetic protein
BP	bisphosphonate
CTGF	connective tissue growth factor
Ctsk	cathepsin K
Coll	collagen
CXCL	C-X-C motif chemokine ligand
CXCR4	C-X-C chemokine receptor type 4
EMT	epithelial to mesenchymal transition
ET-1	endothelin-1
FGF	fibroblast growth factor
Ga	gallium
GM-CSF	granulocyte macrophage colony stimulating factor
HFG	halofuginone
HSCs	hematopoietic stem cells
IGF	insulin-like growth factor
IL	interleukin
LPA	lysophosphatidic acid
miRNAs	microRNAs
MMP	matrix metallo protein
MSC	mesenchymal stem cells
MSG	metastases suppressor gene
MTA1	metastasis- associated protein 1
NGF	nerve growth factor
OPG	osteoprotegerin
OPN	osteopontin
PDGF	platelet -derived growth factor
PgE2	prostaglandin E2
PMMA	poly[methyl methacrylate]
PPi	pyrophosphate
PTEN	phosphatase and tensin homolog
PTHrP	parathyroid hormone-related peptide
RANKL	receptor activator of nuclear factor—κB receptor ligand
SCP	single cell progeny
SDF-1	stromal-derived growth factor-1 (/CXCL12)
siRNAs	small interfering RNAs
SRE	skeletal-related event
TCR	T cell receptor
TGFβ	transforming growth factor-beta
TNFα	tumor necrosis factor-alpha
VEGF	vascular endothelial growth factor
VLA4	very late antigen 4
